# Undiagnosed Abdominal Term Pregnancy with Good Neonatal Outcome

**DOI:** 10.1155/2019/2460485

**Published:** 2019-10-22

**Authors:** Phoy Cheng Chun, Ka-Hee Chua, Mohamed Siraj Shahul Hameed, Manisha Mathur, Wai Kheong Ryan Lee

**Affiliations:** Department of Obstetrics and Gynecology, Kandang Kerbau Women's and Children's Hospital, 229899, Singapore

## Abstract

Abdominal pregnancy is a rare occurrence and it represents only 1% of ectopic pregnancy. We report a case of an abdominal pregnancy that resulted in a term live baby. Diagnosis is unsurprisingly difficult in advanced gestation. A high index of suspicion detailed clinical and imaging examinations are needed to make the diagnosis. Multidisciplinary team involvement is crucial in the management of abdominal pregnancy.

## 1. Introduction

Ectopic pregnancy is a rare occurrence and represents 1–2% of overall pregnancy [1]. 95% of ectopic pregnancy involves the fallopian tube. Abdominal pregnancy represents only 1% of ectopic pregnancy [1], in which the implantation occurs within the peritoneal cavity, outside the fallopian tube and ovary. The incidence rate of abdominal pregnancy is estimated to be 1 : 10000 [1]. It is important to identify such pregnancies as they carry higher mortality and morbidity for both the mother and baby. We report a case of an abdominal pregnancy that resulted in a term live baby.

## 2. Case Report

The patient, a 31-year-old Malay lady, Gravida 2 Para 0 + 1 presented at 38 + 6 weeks of pregnancy with constant lower abdominal pain for 1 day duration associated with back pain.

She had a history of right ectopic pregnancy 7 years ago and was treated with single dose methotrexate. For her current pregnancy, she presented at 35 weeks of gestation for her first antenatal visit. A trans-abdominal (TA) ultrasonography was performed and confirmed a single viable foetus in breech presentation with head circumference (HC) of 309 mm and femur length (FL) of 61 mm, corresponding to 35 weeks and 33 weeks of gestation, respectively. Clinically, her symphysio-fundal height (SFH) was 35 cm. She defaulted her subsequent antenatal appointments.

On arrival, her vitals were stable. Her abdomen was soft with palpable contractions. SFH was 38 cm, longitudinal lie with breech presentation. Speculum examination revealed a closed cervix.

A bedside TA ultrasonography showed a live foetus in breech presentation, HC and FL corresponding to 37 weeks of gestation and AC corresponding to 38 weeks of gestation with estimated foetal weight of 3.3 kg, placenta low covering internal orifice of uterus, and a cervical fibroid measuring 7.6 cm. A repeat TA ultrasonography performed by a dedicated obstetrics sonographer revealed similar findings. Patient was then consented for caesarean section, keeping in view hysterectomy. Preoperative haemoglobin (Hb) was 10.6 g/dL. Four pints of packed red cell (PCT) were arranged.

**Figure 1:**
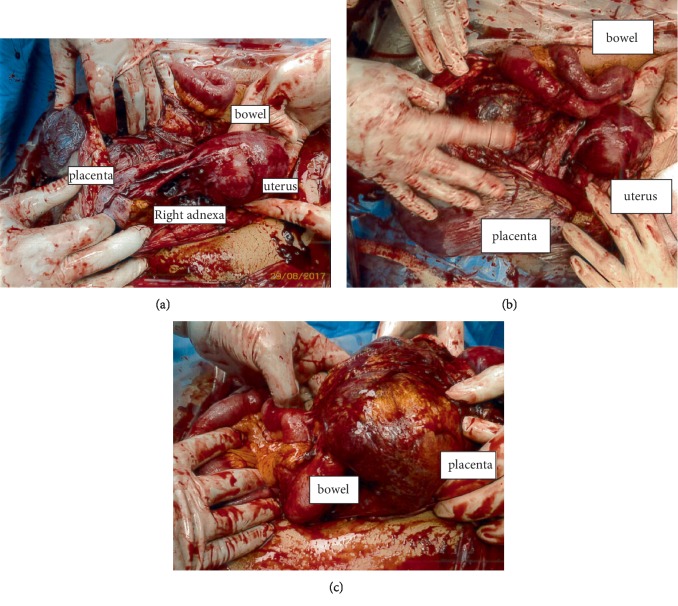
The placenta is located outside of the uterus, attached to the right adnexa, terminal ileum, proximal caecum, and appendix.

**Figure 2:**
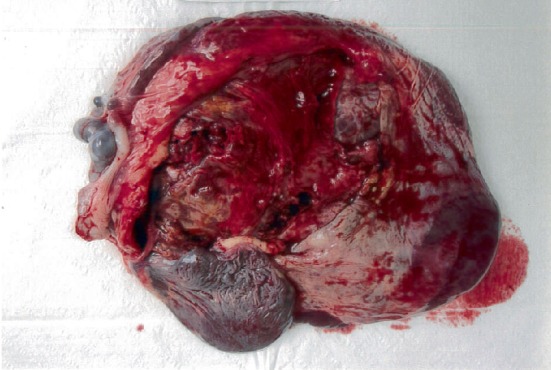
Incomplete placenta. Parts of placenta membrane were left in-situ to avoid bowel injury.

**Figure 3:**
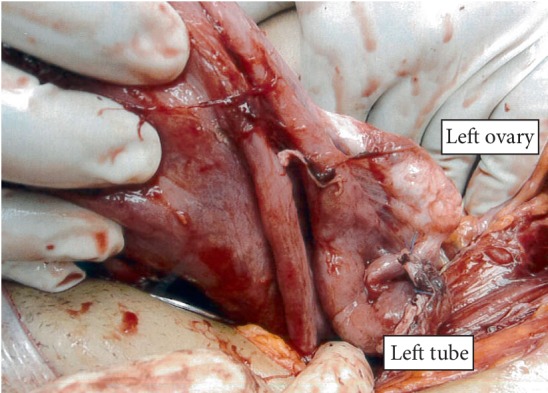
Left fallopian tube and left ovary were identified.

Patient was placed on regional anaesthesia (combined spinal epidural). A Pfannenstiel skin incision was made and her uterus was found to be intact and small upon entering her abdomen. The foetus was found in her abdomen surrounded by a large amniotic membrane filled with clear liquor. The amniotic membrane was incised, and the foetus was delivered cephalic. The foetal Apgar scores were 9 and 9 at one and five minutes, respectively, with birth weight of 2821 gram. The placenta was attached to the right adnexa, terminal ileum, proximal caecum, and appendix (Figure 1(a)–1(c)). Placenta attachments were dissected and ligated, starting from the adnexa and lastly the bowel. Parts of placenta membrane were left in-situ to avoid bowel injury (Figure 2). The left fallopian tube and ovary were seen with the left tube fimbriae end attached to placenta. This was dissected and the left fallopian tube was preserved (Figure 3). The right fallopian tube and ovary were not seen despite a thorough search. There was bleeding from the right uterine vessels, and they were ligated. Intravenous therapy tranexamic acid 1 g was given. Estimated blood loss was 2 litres with Hb of 6.6 g/dL. Two pints of PCT was transfused intraoperatively. Her vitals remained stable. Abdominal redivac drain size #12 was inserted. The duration of operation was 100 min. She was transferred to Intensive Care Unit (ICU) after operation. Posttransfusion Hb was 11.2 g/dL and coagulation profile was normal. Her urine output and vitals remained stable throughout her stay in hospital. Drain output was minimal. Beta HCG 2 days postdelivery was 3617.4 IU/L. She was discharged home on postoperative day 3. Her baby did not require ICU or high dependency unit admission after birth. Placenta histology revealed an intra-abdominal implantation with villous ischemic change and infarction; fimbriae of fallopian tube was seen in specimen; however, ovary was not seen. Patient defaulted follow up after discharge from hospital.

## 3. Discussion

Advanced abdominal pregnancy is extremely rare. Most of the abdominal pregnancy cases are secondary, where a tubal pregnancy ruptures and implants in the peritoneal cavity [2]. In this case, the pregnancy was likely secondary to an undiagnosed ruptured right fallopian tube as placenta histology showed fimbriae end of fallopian tube. Of note, patient had a previous right ectopic pregnancy which was treated conservatively with methotrexate.

Clinical diagnosis and ultrasonography can be difficult at advanced gestation. A diagnosis of extrauterine abdominal pregnancy is frequently missed, with only 45% diagnosed during antenatal period and the rest are diagnosed during surgery [3]. Patients typically have persistent abdominal and/or gastrointestinal symptoms, which were not present in our patient other than the abdominal pain at 38 weeks of gestation.

Sonography is the most effective method in diagnosing abdominal pregnancy. There is report by Radhakrishnan that MRI is a useful adjunct tool to locate the placenta and delineate its origin and attachments as well as confirm the diagnosis. Perioperative angiogram might be useful in mapping the vascular supply of the placenta preoperatively, also, embolization or balloon obturation of vessels to control haemorrhage [4].

Major criteria for diagnosis of intra-abdominal pregnancy listed by Allibone et al. include: (1) demonstration of a foetus in gestational sac outside the uterus, or the depiction of an abdominal or pelvic mass identifiable as the uterus separate from the foetus, (2) failure to see a uterine wall between the foetus and the urinary bladder, (3) recognition of a close approximation of the foetus to the maternal abdominal wall, and (4) localization of placenta outside the confines of the uterine cavity [5]. Other additional criteria include oligohydramnios, abnormal foetal lie, placenta praevia appearance, and maternal bowel gas impending foetal visualization. On retrospective review of the ultrasound images, there was indeed no uterine wall between the foetus and urinary bladder, and the placenta appeared to be praevia. However, these signs were not picked up prior to Caesarean section.

Stevens (1993) reported a 21% birth defect risk associated with extra-uterine pregnancies, which include, central nervous system malformations, facial and cranial asymmetry, joint abnormalities and limb defects [6]. The lack of amniotic fluid buffer leading to foetal compression may be the cause of these defects. The patient's baby was examined by neonatology team and no malformation was found on the baby upon delivery.

One of the major concerns intraoperatively was the management of placenta. Bleeding from placenta implantation site can be life-threatening especially if the blood supply cannot be identified or ligated completely. Expectant management can be considered in such situation. By leaving the placenta in situ, blood loss and blood transfusion requirement are less compared to removal of placenta [7]. However, this approach can be associated with postoperative morbidity and mortality such as peritonitis, abscess formation, and prolonged hospital stay [8]. Till date, discussion on this issue is still ongoing. Careful assessment should be carried out when deciding the suitable approach for each patient. In this case, the blood supply to the placenta was identified and almost all of the placenta was removed with minimal placenta tissues attached to the bowel. Human chorionic gonadotropin (HCG) level was measured postsurgery, with a plan of repeat HCG level during postnatal follow up in outpatient clinic.

## 4. Conclusion

Abdominal pregnancy with resultant healthy live newborn is a rare occasion. Diagnosis is unsurprisingly difficult in advanced gestation. A high index of suspicion, detailed clinical and imaging examinations are needed to make the diagnosis.

In addition, the surgeon's experience and clinical judgment, co-operation with the anaesthetists and neonatologist teams, availability of blood products for potential massive blood transfusions, immediate detection, and rapid surgical repair help to minimise life-threatening complications.
